# Application of Machine Learning Algorithms to the Discretization Problem in Wearable Electrical Tomography Imaging for Bladder Tracking

**DOI:** 10.3390/s23031553

**Published:** 2023-01-31

**Authors:** Bartłomiej Baran, Edward Kozłowski, Dariusz Majerek, Tomasz Rymarczyk, Manuchehr Soleimani, Dariusz Wójcik

**Affiliations:** 1 Research & Development Centre Netrix S.A., 20-704 Lublin, Poland; 2 Faculty of Management, Lublin University of Technology, 20-618 Lublin, Poland; 3 Faculty of Fundamentals of Technology, Lublin University of Technology, 20-618 Lublin, Poland; 4 WSEI University, 20-209 Lublin, Poland; 5 Department of Electronic and Electrical Engineering, University of Bath, Bath BA2 7AY, UK

**Keywords:** electrical tomography, sensors, numerical calculation, machine learning, elastic net, logistic regression, decision trees, discriminant analysis, image reconstruction

## Abstract

The article presents the implementation of artificial intelligence algorithms for the problem of discretization in Electrical Impedance Tomography (EIT) adapted for urinary tract monitoring. The primary objective of discretization is to create a finite element mesh (FEM) classifier that will separate the inclusion elements from the background. In general, the classifier is designed to detect the area of elements belonging to an inclusion revealing the shape of that object. We show the adaptation of supervised learning methods such as logistic regression, decision trees, linear and quadratic discriminant analysis to the problem of tracking the urinary bladder using EIT. Our study focuses on developing and comparing various algorithms for discretization, which perfectly supplement methods for an inverse problem. The innovation of the presented solutions lies in the originally adapted algorithms for EIT allowing for the tracking of the bladder. We claim that a robust measurement solution with sensors and statistical methods can track the placement and shape change of the bladder, leading to effective information about the studied object. This article also shows the developed device, its functions and working principle. The development of such a device and accompanying information technology came about in response to particularly strong market demand for modern technical solutions for urinary tract rehabilitation.

## 1. Introduction

Lower urinary tract diseases are on the rise and reduce the quality of life of those affected. It is estimated that up to 50% of the population will suffer from various forms of incontinence at some stage in their lives [1,2]. In some cases, there will be a spontaneous regression, but approximately 70% of this group will develop persistent urinary incontinence of varying severity. Approximately 50% of people with incontinence use pads only to prevent leakage and soiling of underwear and do not attempt self-treatment. In 15% of patients with severe urinary incontinence, surgical treatment is resorted to, which improves the quality of life to varying degrees. However, according to the literature in the field of functional disorders of the urinary system [3,4], it is argued that the rehabilitation of the muscles responsible for the efficient emptying of the urinary tract should be performed both before and after a surgical procedure. Nowadays, the treatment of bladder dysfunction based on biofeedback and EMG therapy is a universal standard. However, the devices currently available on the market struggle with poor comfort and mobility, which does not allow for daily and regular rehabilitation. In contrast to static imaging with ultrasound, CT or nuclear magnetic resonance, EIT allows continuous (up to a dozen hours) diagnostics of urinary tract function. The great advantages of this method are its low invasiveness, long-term evaluation of urinary tract function and the relatively low price of performing the test [5,6,7,8,9,10].

**Figure 1:**
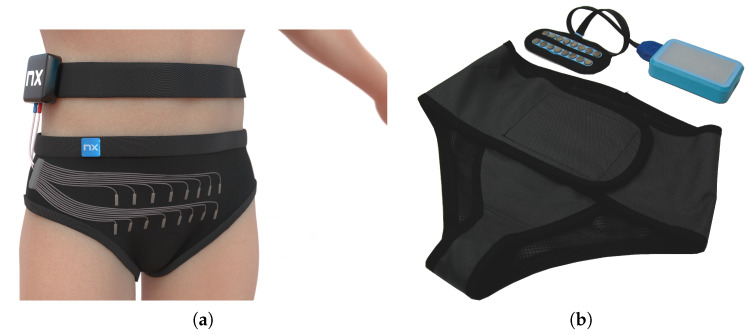
Panel (**a**) shows a visualization of an innovative device for measuring EIT and EMG with a module for muscle electrostimulation. Panel (**b**) presents a prototype of a wearable EIT measurement system with skin-safe sensors and a comfortable to-carry recording device.

To face the aforementioned problems, we developed a device for non-invasive monitoring and diagnosis of lower urinary tract functional disorders, as presented in Figure 1. The device will enable the measurement of muscle tension (EMG) with the possibility of electrostimulation and biofeedback-type therapy. At the same time, the device has implemented a system for visualization of the urinary tract based on EIT and computational intelligence algorithms. Due to its compact size, the device can be used in specialized healthcare centers and at home.

In this article, we briefly introduce the concept of the developed device equipped with the EIT imaging technique. Such a device can be equipped with optional EMG diagnostics of muscle and nerve function or electrostimulation to allow for the required muscle contractions. Firstly, we describe the principle of operation of the EIT system and the equipment required to create such a device. Then, we present and compare various mathematical algorithms for discretizing FEM elements. In principle, the main idea is to construct a classifier allowing for assigning the FEM components to a class consisting of the inclusion or background elements. Our study focuses on the reconstruction of the urinary bladder with EIT using experimental data and applying machine learning algorithms. Similar studies have been conducted in this field, but the results were obtained with a simulated data set [5,6,11], or reconstruction has been obtained with measurement data using the Gauss–Newton method [12], known for its low-quality results with experimental data frames.

## 2. Materials and Methods

EIT is an imaging technique that exploits the different electrical properties of materials [13,14,15,16,17,18,19,20,21,22,23,24,25]. In this method, a source of electric voltage is connected to an object, resulting in current flows through its interior and electrical potential distributions at the surface of that subject item. The gathered information is processed by an algorithm that reproduces the internal impedance distribution. Such a method has a relatively low spatial image resolution. The difficulty in obtaining high resolution is primarily due to the limited number of measurements, the nonlinear current flow through the studied medium and the insufficient sensitivity of the voltage measurement apparatus to changes in conductivity in a given area. Image reconstruction is very sensitive to pervasive modeling errors, which are caused by inaccurately derived auxiliary variables of the measurement model. In practice, first and foremost, if the shape of the object is inaccurately known, errors in its modeling cause divergence from the real object. From a mathematical point of view, EIT belongs to the category of inverse problems designed to study the distribution of electromagnetic fields. An inverse problem is a procedure of traceability, development or synthesis in which the parameters describing the electrical field are predicated based on having some information specific to it. Such problems are challenging and cumbersome to analyze. This is because there is usually not enough or a redundancy of information, which is sometimes contradictory or linearly dependent. The numerical examination of the physical problem based on EIT is solved using the finite element method (FEM).

### 2.1. Measurement System

The device presented in Figure 1b is equipped with 16 channels for measuring the surface potential of the body with an accuracy of 1 μV. Each of the channels is independently programmable (measurement and stimulation parameters), allowing one to perform a current simulation arbitrarily. During a single measurement, two of the attached electrodes are used as charge injectors forcing the flow of AC current of constant amplitude through the object under examination, while the remaining electrodes work as measuring electrodes probing electrical voltages at points of contact with the object. After data are collected from one sampling, the injecting electrodes are switched to the next in the sequence, and the procedure repeats until data are collected from all specified combinations. The developed prototype is a compact, single-board EIT scanner capable of performing measurements on 16 electrodes in any desired configuration. In our setup, the electrodes are arranged in two rows of eight electrodes, which allow for 2D (8 sensors) or 3D (16 sensors) reconstruction by solving the inverse problem. The stimulation between injecting electrodes is performed with a current in mA range at 100 kHz. The injection appears only between electrodes from the same row. As a result, the device records 256 different voltages in a single attempt.

**Figure 2:**
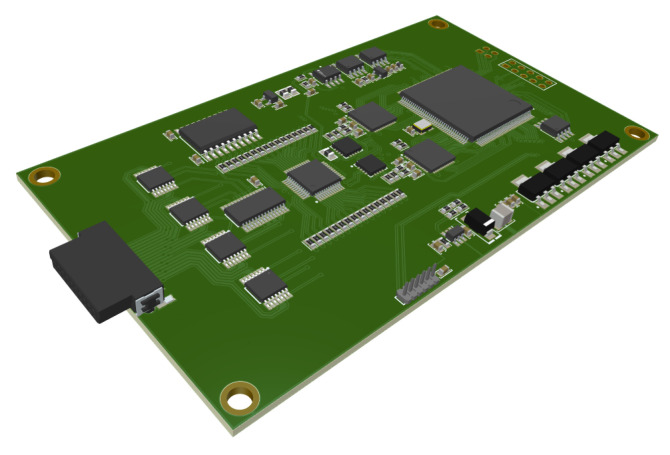
The main board with a programmable logic controller (PLC) used in the EIT device.

**Figure 3:**
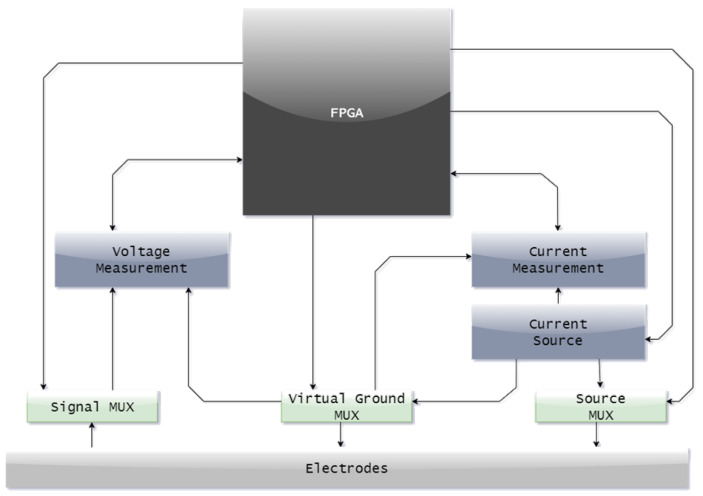
The block diagram of a solution containing a field-programmable gate array (FPGA).

In detail, the device consists of a current source, a current measurement module, a voltage measurement module, a set of multiplexers and a control unit. The central unit presented in Figure 2 is designed to fit into a modest ergonomic case and constructed with multilayer circuits and assembled with BGA soldering. The block structure of this solution is presented in Figure 3. The power source is connected to any electrode pair via an analog dual 16-channel multiplexer. It consists of two independent digital-to-analog converters where the voltage output of one of the converters generates the shape of the forced current waveform, and the voltage output of the other is the reference voltage. The converters are controlled from the FPGA via a serial bus. Such a solution allows precise control of the waveform shape and its amplitude. The current flow is tested on the measurement resistor using a high-end ADC along with the necessary signal conditioning circuits. The circuit has programmable gain, pre-filtering, and differential signal-forming capability. The ADC sends the data to the FPGA via a parallel bus. Based on the reading of the value from the converter, the logic regulates the value of the current obtained from the current source. The electrode voltages are tested with a measurement module consisting of a series of signal conditioning circuits and an analog-to-digital converter similar to current measurement. The electrodes are connected directly to a set of preamplifiers, followed by a 16-channel multiplexer. The multiplexed signal is transferred to the amplifier with a programmable enhancement, which is equipped with an additional module that enables the signal conversion to a differential signal. The next step transfers the signal to an analog-to-digital converter through a filter. The control unit in the device is an Altera Cyclone IV FPGA with 144 leads and 10,000 logic elements. It controls all processes in the measurement cycle and is used for data acquisition and transfer. The electronics and housing are designed to fulfill the standard of EMC requirements. It was ensured that the electronics did not have a negative impact on the surroundings and were not exposed to electrostatic discharges from the environment.

This high-end 16-bit digital-to-analog converter has a sampling rate of up to 25 Msps, a signal conditioning circuit containing a measurement amplifier with adjustable gain and a differential amplifier that adjusts the signal under test to the inputs of the ADC accordingly. The main component of the measurement part is the LTC2202 analog-to-digital converter, a 16-bit ADC with a sampling frequency of 10 Msps. This chip has a built-in PGA front-end that allows changing the input range 1× or 1.5×, is powered by a single 3.3 V voltage and has a high-speed parallel interface. A signal conditioning circuit consisting of a measurement amplifier and a differential amplifier has been used at the input of the transmitter, allowing 1–100× gain adjustment and signal adjustment to the differential input of the transmitter. Digital gain control is realized with a digital potentiometer in an adjustable resistor circuit with a resolution of 10-bit.

The device supports communication with a master device via Bluetooth or Wi-Fi and integrates with PCs running the Microsoft Windows operating system and mobile devices with Google Android or Apple iOS. The device is equipped with software for superior devices that performs the function of control and programming of devices as well as visualization and archiving of test results. An optional module with a touch screen allows you to control the operation of the device and change its settings without the need to use a computer. This solution will be equipped with mechanisms allowing for remote control of rehabilitation settings and control of its results.

### 2.2. Measurement Data Acquisition

**Figure 4:**
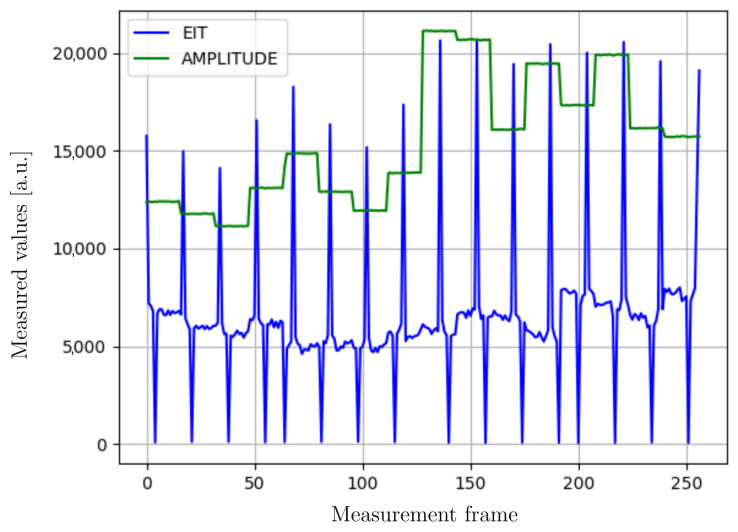
An example of a measurement data frame. The data frame consists of 256 components of the recorded EIT voltages and the applied voltage for the injection current (marked with a green line).

The measurement data are collected and aggregated by the device, then transmitted as data packets to an external unit. Figure 4 shows an example of a measurement obtained on a patient under study. It shows voltages on the body surface (blue line) and corresponding electrical potential differences between injection and ground electrodes (green line). This frame presents measurement data recorded from 16 measurement electrodes. When the device is connected to a computer via the USB port, the delay in communication is close to 6 ms; using wireless communication, the delay in sending commands is around 50 ms. At the current stage of development, the designed device is able to perform 2.5 measurements per second.

## 3. Discriminant Algorithms

This section includes descriptions of various mathematical models used to solve the discrimination problem. We begin by defining the problem of the discrete dataset consisting of the mesh elements representing the torso cross section at the urinary bladder level. For the mesh, a dataset was prepared from the solution of the forward problem, assuming different shapes and positions of inclusions. As a result, the dataset contains 5000 different examples of simulated measurements.

In the further section, we concentrate on the description and theoretical aspects of the implemented methods. We present the algorithms based on the linear regression supported by elastic net regularization, the linear, quadratic and regularized discriminant analysis with the possibility of application of principal component analysis (PCA). Moreover, we include a short description of the decision tree method.

### 3.1. The Finite Element Mesh and Discretization Problem

**Figure 5:**
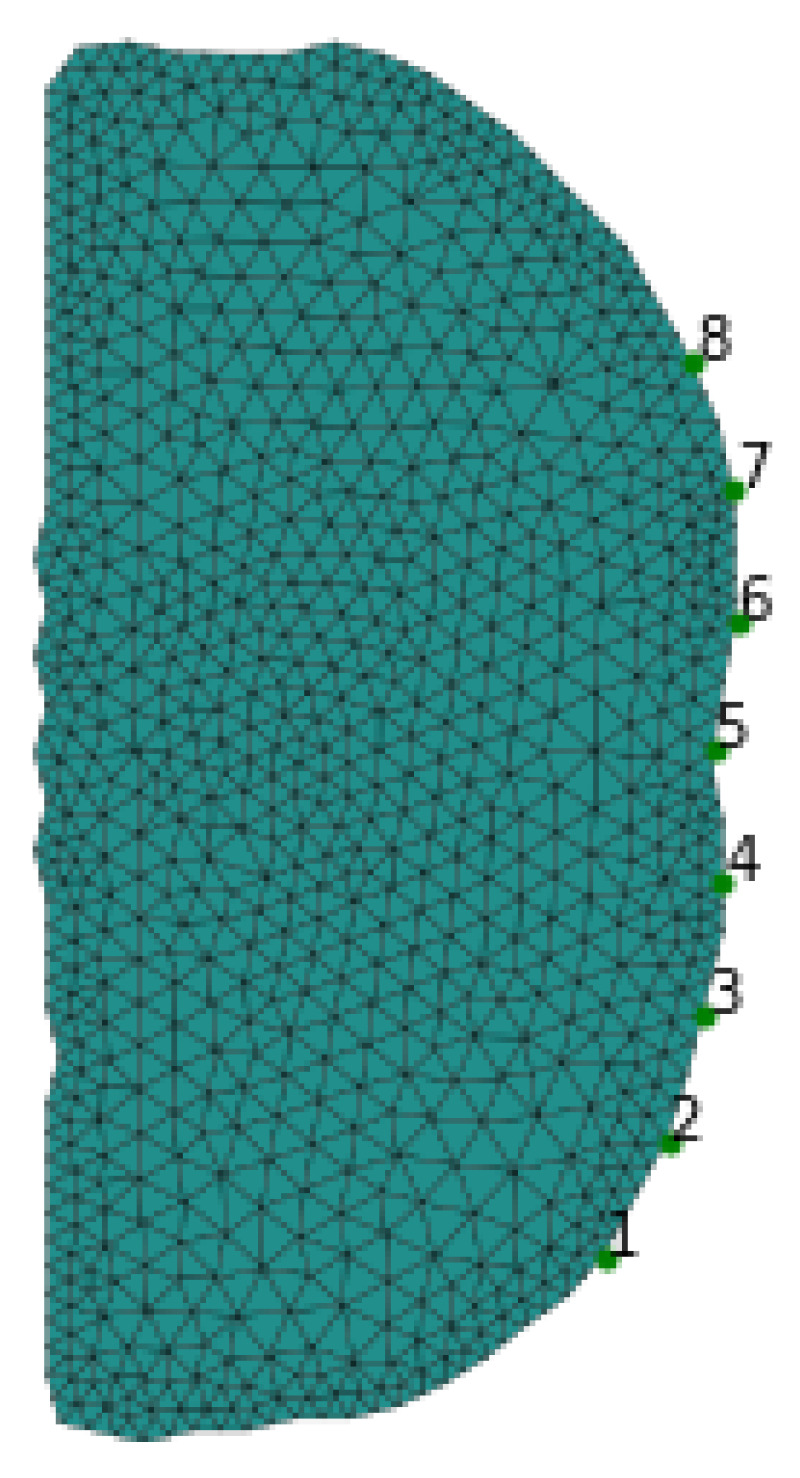
The finite element mesh of the torso cross section at the urinary bladder level.

The main purpose of discriminant analysis (pattern classification) is to find a classification rule [13,14]. This process involves identifying eligibility for a certain class based on observations of the independent variable. The decision on class membership is made based on knowledge of the distribution of the independent variable and the distribution of class a priori. In the case of EIT, the elements of the field of view are a collection of such a class. Figure 5 presents the finite element mesh composed of 848 nodes and 1555 triangles. This model uses eight line electrodes that are placed in front of the torso.

Such a discrete set of finite elements with different inclusion positions and shapes is a learning dataset for the models introduced below. This learning dataset can be written as $D=\left\{\left(x_{i},y_{i}\right):x_{i}\in R^{m},\;y_{i}\in \left\{0,1\right\},\;1\leq i\leq n\right\}$. The components of the sequence ${\left\{x_{i}\right\}}_{1\leq i\leq n}$ belong to two classes, where membership to the class is expressed as $y_{i}\in \left\{0,1\right\}$ for 1≤i≤n. In the analyzed case of the presence of an inclusion for a finite element (a pixel in the field of view), we assume $y_{i}=1$, while if the element does not represent an inclusion, we take $y_{i}=0$. Analyzing the signal received from the sensors $x\in R^{m}$, it is possible to classify each finite element. Logistic regression was used to create a classifier (finite element mapping) $f:R^{m}\to \left\{0,1\right\}$.

### 3.2. Logistic Regression (LR)

Let $\left(\Omega ,F,P\right)$ be a probabilistic space and *Y* a random variable with a discrete distribution $Y:\Omega \to \left\{0,1\right\}$. Then, the ratio of the probability of success to the probability of failure can be defined as

(1) $$\theta \left(X\right)=\frac{P\left(Y=1\left|X\right.\right)}{1-P\left(Y=1\left|X\right.\right)}.$$

The main purpose of logistic regression (LR) is to evaluate the probability of success $P\left(Y=1\left|X\right.\right)$, where *X* denotes the realization of predictors [13,15,16]. It can be assumed that

(2) $$P\left(Y=1\left|X\right.\right)=p\left(X\right).$$

Knowing that the probability of success is $p\left(X\right)\in \left(0,1\right)$, from Equation (1) it follows that the chance of a success is $\theta \left(X\right)\in \left(0,\infty \right)$, while the *log-odds* (also known as *logit*) are $\ln\theta \left(X\right)\in \left(-\infty ,\infty \right)$.

Using the logistic regression method, we analyze the linear dependence of the logarithm on the independent variables *X*. For this purpose, we examine the correlation specified by the following expression

(3) $$\ln\theta \left(X\right)=\ln\left(\frac{p(X)}{1-p(X)}\right)=X\beta +\varepsilon ,$$

where ε is a random variable with a normal distribution $N\left(0,\sigma^{2}\right)$ and an estimator $\beta =\left(\beta_{1},\beta_{2},\beta_{3},...,\beta_{m}\right)\in R^{m}$.

Using Equation (3), we can derive the probability of success as

(4) $$p\left(\beta ,X\right)\overset{def}{=}p\left(X\right)=\frac{e^{X\beta }}{1+e^{X\beta }}.$$

In order to estimate β parameters, maximum-likelihood estimation (MLE) was used [17].

#### Logistic Regression with Elastic Net Regularization

Due to the collinearity of independent variables (measurements obtained from the sensors), certain regularization methods should be applied. In our work, we focus on elastic net regularization [18], which is a linear combination of LASSO (least absolute shrinkage and selection operator) regression and ridge regression, called Tikhonov regularization [19,20].

To determine the linear regression parameters in the model defined in Equation (3), one needs to solve the following relation

(5) $$\max_{\beta }\left\{\sum_{i=1}^{N}\left(y_{i}x_{\left(i\right)}\beta -\ln\left(1+e^{x_{\left(i\right)}\beta }\right)\right)-\lambda P_{\alpha }\left(\beta \right)\right\},$$

where λ>0, 0≤α≤1 and $P_{\alpha }$ represents the penalty given by the formula

(6) $$P_{\alpha }\left(\beta \right)=\alpha {∥\beta ∥}_{L_{1}}+\frac{1-\alpha }{2}{∥\beta ∥}_{L_{2}}=\sum_{j=1}^{p}\left(\alpha \left|\beta_{j}\right|+\frac{1-\alpha }{2}\beta_{j}^{2}\right).$$

The penalty $P_{\alpha }\left(\beta \right)$ is the linear combination of the norms of the vector of estimators β in $L_{1}$ and $L_{2}$ spaces. For α=0, we have ridge regression, while for α=1 we obtain LASSO regression. In order to adjust the regularization parameters, Equation (5) is solved for each finite element. First, the sequence of possible values of the λ parameter is determined. Then, for different values of the regularization parameter, β coefficients are determined using the K-fold cross-validation method. As λ parameter and β parameter estimators, we take such values for which the cross-validation estimate of error is smallest (in the case under consideration, we chose such values for which the cross-validation estimate of accuracy was highest).

### 3.3. Linear Discriminant Analysis (LDA)

For the given learning dataset *D*, the probabilistic space $\left(\Omega ,F,P\right)$ and random variable *Y*, we can define the a priori distribution for *Y* as

(7) $$\pi_{k}=\frac{n_{k}}{n},$$

where $n_{k}=\#\left\{i:y_{i}=k\right\}$, and $\pi_{0}+\pi_{1}=1$. For $k\in \left\{0,1\right\}$, we construct a decision rule on the basis of Bayes’ theorem

(8) $$P\left(Y=k|X=x\right)=\frac{P\left(X=x|Y=k\right)\pi_{k}}{\sum_{j=0}^{1}P\left(X=x|Y=j\right)\pi_{j}}.$$

Determining class affiliation based on Equation (8), we compare values $P\left(X=x|Y=k\right)\pi_{k}$. A larger value of this product means a higher probability that the random variable *Y* will take the value *k* (i.e., the observation *x* belongs to the *k* class) [13,14,16]. Considering the linear discriminant analysis, we assume that the covariance matrix of the random variable *X* for each group is identical, i.e., $\Sigma_{0}=\Sigma_{k}=\Sigma$. The conditional distribution of random variable *X* belonging to the class *k*, $k\in \left\{0,1\right\}$ is given as a formula

(9) $$f_{k}\left(x\right)=P\left(X=x|Y=k\right)=\frac{1}{{\left(2\pi \right)}^{m/2}\sqrt{\left|\Sigma \right|}}\exp\left(-\frac{1}{2}{\left(x-\mu_{k}\right)}^{T}\Sigma^{-1}\left(x-\mu_{k}\right)\right).$$

To compare the probabilities of belonging to two different classes presented in Equation (8), it is enough to analyze the logarithm of the quotient of these probabilities, i.e.,

(10) $$\log\frac{P\left(Y=1|X=x\right)}{P\left(Y=0|X=x\right)}=\log\pi_{1}-\log\pi_{0}-\frac{1}{2}\mu_{1}^{T}\Sigma^{-1}\mu_{1}+\frac{1}{2}\mu_{0}^{T}\Sigma^{-1}\mu_{0}+x^{T}\Sigma^{-1}\mu_{1}-x^{T}\Sigma^{-1}\mu_{0}.$$

Moreover, for class $k\in \left\{0,1\right\}$, we can introduce a linear discriminant function in the form

(11) $$\delta_{i}\left(x\right)=\log\pi_{i}+x^{T}\Sigma^{-1}\mu_{i}-\frac{1}{2}\mu_{i}^{T}\Sigma^{-1}\mu_{i}$$

and a definition of the plane separating the two classes as

(12) $$H=\left\{x\in R^{m}:P\left(Y=1|X=x\right)=P\left(Y=0|X=x\right)\right\}.$$

Combining Equations (10) and (11), we obtain the plane *H*

(13) $$H=\left\{x\in R^{m}:\delta_{1}\left(x\right)=\delta_{0}\left(x\right)\right\},$$

which splits the entire space $R^{m}$ into two separable sets, where the membership of an observed signal in a set is equivalent to membership in the corresponding class. Based on the introduced properties, the decision rule can be expressed in the form

(14) $$\hat{Y}=\left\{\begin{array}{cc} 1, & \delta_{1}\left(x\right)\geq \delta_{0}\left(x\right),\\ 0, & \delta_{1}\left(x\right)<\delta_{0}\left(x\right) \end{array}\right.=\;\underset{k\in \{0,1\}}{argmax}\;\delta_{k}\left(x\right).$$

As the estimators of the unknown parameters of the distributions of observations for each class, we determine:

- expected values
  $${\hat{\mu }}_{k}=\frac{1}{n_{k}}\sum_{i:y_{i}=k}x_{i}$$
  for $k\in \left\{0,1\right\}$;
- covariance matrix
  $$\hat{\Sigma }=\frac{1}{n-2}\sum_{k=0}^{1}\sum_{i:y_{i}=k}\left(x_{i}-\hat{\mu_{k}}\right){\left(x_{i}-\hat{\mu_{k}}\right)}^{T}.$$

It is important to remember that in the EIT experiment, the predictors are highly correlated, so to overcome the problem of the singularity of the Σ matrix, it is necessary to use regularization techniques [21,22].

### 3.4. Quadratic Discriminant Analysis (QDA)

Quadratic discriminant analysis (QDA) presents an alternative approach to defining the classification rule. Identical to the LDA, we assume that the conditional distribution $f_{k}\left(x\right)=P\left(X=x|Y=k\right)$ of a random variable *X* is a normal distribution $N\left(\mu_{k},\Sigma_{k}\right)$ for $k\in \left\{0,1\right\}$. The difference is that for LDA we assume the identical covariance matrix of the random variable *X* for each group, i.e., $\Sigma_{0}=\Sigma_{1}=\Sigma$, while in the case of QDA, they are different. Therefore, for each class we determine the expected value vector and covariance matrix:

(15) $${\hat{\mu }}_{k}=\frac{1}{n_{k}}\sum_{i:y_{i}=k}x_{i},$$

(16) $$\hat{\Sigma_{k}}=\frac{1}{n_{k}-1}\sum_{i:y_{i}=k}\left(x_{i}-\hat{\mu_{k}}\right){\left(x_{i}-\hat{\mu_{k}}\right)}^{T},$$

where $k\in \left\{0,1\right\}$.

Comparing the probabilities of belonging to two different classes in Equation (8), we analyze the logarithm of the quotient of these probabilities, i.e.,

(17) $$\begin{array}{ccc} \log\frac{P\left(Y=1|X=x\right)}{P\left(Y=0|X=x\right)} & = & \log\pi_{1}-\frac{1}{2}{\left(x-\mu_{1}\right)}^{T}\Sigma_{1}^{-1}\left(x-\mu_{1}\right)-\frac{1}{2}\log\det\left(\Sigma_{1}^{-1}\right)\\ & & -\log\pi_{0}+\frac{1}{2}{\left(x-\mu_{0}\right)}^{T}\Sigma_{0}^{-1}\left(x-\mu_{0}\right)+\frac{1}{2}\log\det\left(\Sigma_{0}\right) \end{array}$$

Just as previously for LDA, we can define a quadratic discriminant function $\delta_{i}\left(x\right)$ for QDA and class $k\in \left\{0,1\right\}$ in the following form

(18) $$\delta_{i}\left(x\right)=\log\pi_{k}-\frac{1}{2}{\left(x-\mu_{k}\right)}^{T}\Sigma_{k}^{-1}\left(x-\mu_{k}\right)-\frac{1}{2}\log\det\left(\Sigma_{k}^{-1}\right).$$

For QDA, the decision rule is expressed by Equation (14).

### 3.5. Regularized Discriminant Analysis

When predictors are highly correlated with each other, the prediction with LDA and QDA models is unstable. Friedman proposed a compromise (in the sense of co-integration) between LDA and QDA that allows the covariance for QDA models to shrink toward LDA [23]. Such a technique is called regularization, while the method is referred to as RDA (regularized discriminant analysis). This method is very similar to ridge regression, allowing for shrinkage of covariances between characteristics, namely the covariance matrices for each class are referred to as

(19) $$\Sigma_{k}^{\alpha }=\alpha \Sigma_{k}+\left(1-\alpha \right)\Sigma$$

for $k\in \left\{0,1\right\}$ and $\alpha \in \left[0,1\right]$.

In practice, the α parameter is chosen so that the classification error when applying the model to validation data (or using cross-validation) is as small as possible. For LDA models, the identical regularization technique can also be used; the covariance matrix is determined by the formula

(20) $$\Sigma^{\gamma }=\gamma \Sigma +\left(1-\gamma \right)\sigma^{2}I,$$

where $\gamma \in \left[0,1\right]$ and $I\in R^{m\times m}$ is an identity matrix. In the results section, the RDA was used in the discrimination, where Equation (20) was used to shrink the covariance matrix.

### 3.6. Principal Component Analysis (PCA)

As already described, our X signals can be highly correlated. Another method of eliminating redundancy could be principal component analysis. It relies on identifying the factors (components) present in a dataset by creating linear combinations of the original variables in such a way that the new components explain the largest part of the variation in the original space [13,24,25]. We call the coordinates of the new system loads of the created principal components. In the new auxiliary space, most variability is explained by the initial factors. PCA is often used to reduce the size of a statistical dataset by discarding the last factors [15].

### 3.7. Decision Trees

A decision tree is a hierarchical structure representing a classification or regression model. They are used especially often when the functional form of the correlation between predictors and the outcome variable is unknown or hard to determine. Each decision tree consists of a root, nodes and leaves. The root is called the initial node of the tree, from which subsequent descendant nodes are formed through divisions. The terminal nodes that do not undergo divisions are called leaves, and the lines connecting the nodes are called branches.

If the tree is used for classification tasks, the leaves contain information about which class in a given sequence of subdivisions is most likely to occur. On the other hand, if the tree is for regression purposes, the leaves contain conditional measures of the outcome’s central tendency (usually the mean). The condition represents a series of divisions leading to a given terminal node (leaf). In both cases (classification and regression), the tree tends to such a division that successive nodes and leaves are as homogeneous as possible regarding the outcome variable.

There are a variety of types of splitting rules used in decision trees. They are selected so that subsequent nodes are characterized by less impurity. In classification trees, the most commonly used measure of impurity is the Gini index

(21) $$Gi_{T_{n}}\left(c|t\right)=\sum_{x\in R_{t}}\frac{|T_{n,t=r}|}{|T_{n}|}Gi_{T_{n,t=r}}\left(c\right),$$

where

(22) $$Gi_{T_{n,t=r}}\left(c\right)=\sum_{d\in C}P_{T_{n,t=r}}\left(c=d\right)\;\cdot \;\left(1-P_{T_{n,t=r}}\left(c=d\right)\right)=1-\sum_{d\in C}P_{T_{n,t=r}}^{2}\left(c=d\right).$$

Among the biggest advantages of using decision trees are:

- Easy to interpret;
- Do not require tedious data preparation (no standardization, introduction of binary variables, allows for missing data);
- Potential non-linearity of the relationship between the outcome variable and the predictors;
- Robust to deviations from assumptions;
- Allows for a quick analysis of large data sets.

The biggest disadvantage of single decision trees is low predictive power, especially in complex tasks. In such cases, it is recommended to use tree ensembles in the form of random forests, bagging or boosting.

### 3.8. Measures of Fit Assessment

For each element in the dataset *D*, based on the readings of *X*, we determine the probability of inclusion $P\left(Y=1|X\right)$ based on model prediction, assuming

(23) $$\mathrm{Belonging}\;\mathrm{to}\;\mathrm{the}\;\mathrm{area}=\left\{\begin{array}{cc} \mathrm{inclusion}, & P\left(Y=1|X\right)\geq t\\ \mathrm{background}, & P\left(Y=0|X\right)<t \end{array}\right.$$

for the threshold $t\in \left(0,1\right)$. The most commonly accepted threshold value is 0.5. The elementary terminology and factors describing the recognition of inclusions in the field of view are presented below. We take the lack of inclusion in a finite element location as a negative case (N), while the presence of inclusion is a positive case (P). The confusion matrix should be specified with the following values: *TP* (true positive), the number of finite elements for which inclusions were correctly recognized; *TN* (true negative), the number of finite elements for which the lack of inclusion was correctly identified; *FP* (false positive), the number of elements without inclusions, which are assigned to have inclusions (false alarm); *FN* (false negative), the number of finite elements with inclusions, for which they were considered to have no inclusions (see Table 1).

We use the standard definition of basic fit measures as follows [26]:

(24) $$Accuracy=\frac{TP+TN}{TP+TN+FP+FN},$$

(25) $$TruePositiveRate=Sensivity=\frac{TP}{TP+FN},$$

(26) $$Specificity=1-FalsePositiveRate=\frac{TN}{TN+FP},$$

(27) $$PositivePredictiveValue=\frac{TP}{TP+FP},$$

(28) $$NegativePredictiveValue=\frac{TN}{TN+FN},$$

(29) $$Prevalence=\frac{TP+FN}{TP+TN+FP+FN},$$

(30) $$DetectionRate=\frac{TP}{TP+TN+FP+FN},$$

(31) $$DetectionPrevalence=\frac{TP+FP}{TP+TN+FP+FN},$$

(32) $$BalancedAccuracy=\frac{Sensivity+Specificity}{2}.$$

The use of so many measures of model fit was dictated by the fact that each measure exposes a different aspect of model fit. Using them complementarily helps assess the model’s performance.

In the EIT image reconstruction, it is also necessary to describe the ability to find inclusions in the field of view. To evaluate the ability of the classifier based on the logistic regression (see e.g., [26,27]), we determine the curve describing the receiver operating characteristic (ROC curve). This curve illustrates the relationship between sensitivity and specificity for different threshold levels. The diagonal in the ROC figure describes a strategy based on guessing inclusions during reconstruction. When the ROC is above the diagonal, it means that the recognition technique is clearly better than guessing. The area under the ROC curve in the literature is called AUC (area under ROC curve) and denotes a measure of predictability. This quantity is also included in the tables describing the reconstructions.

To determine the credibility of performed discretizations (consistency between the inclusion and prediction), we rely on Cohen’s ratio κ defined as follows:

(33) $$\kappa =\frac{2(TP\;\cdot \;TN-FN\;\cdot \;FP)}{\left(TP+FP)(FP+TN)+(TP+FN)(FN+TN\right)},$$

where κ∈[0,1]. A larger value of κ determines greater consistency between inclusion and discretization results.

On the other hand, to verify an inconsistency between inclusion and discretization result, we use McNemar’s test defined as follows:

(34) $$\chi^{2}=\frac{{\left(FP-FN\right)}^{2}}{FN+FP},$$

This statistical test compares the sensitivity and specificity of discretization result.

## 4. Results

In this section, we present the results of the model classification and the tables with the measures of fit describing the performance of the acquired discretizations. For all of the specified methods, we present the example of the result obtained for different regularization approaches and compare the inclusion pattern corresponding to the used simulated data frame.

**Figure 6:**
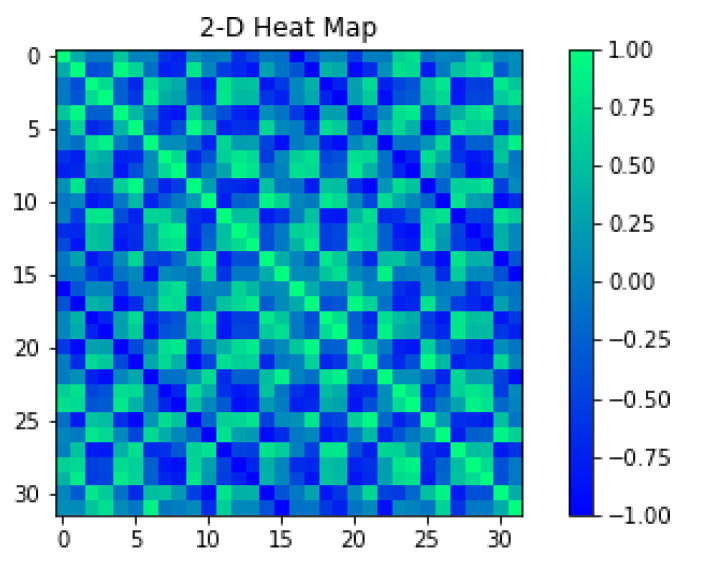
The heatmap of the covariance matrix showing correlations between predictors.

The single data frame consists of 32 elements for 2D simulated voltage at the edge of the model presented in Figure 5. Due to the fact that in EIT the measurements of the predictors are highly correlated, we present a covariance matrix in Figure 6 confirming this statement.

In the last part of this section, we present the application of prepared algorithms to the case of a healthy male in his 20s with an almost full bladder. We show a comparison of results for all introduced discretization methods.

### 4.1. Results for Logistic Regression

**Figure 7:**
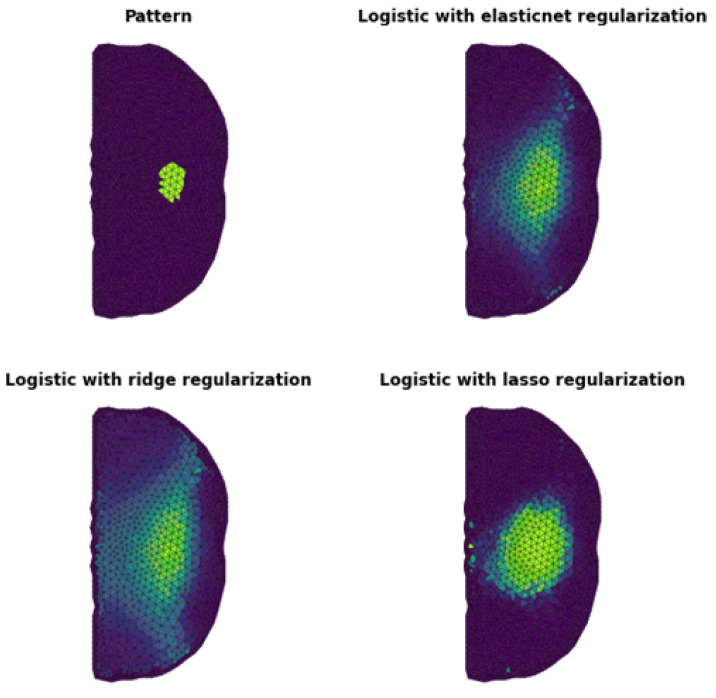
The top left panel shows the shape of the inclusion. The other panels show the results obtained by logistic regression with regularization methods as shown in the graphs.

In Figure 7, we show the results obtained for logistic regression with applied regularization methods, where the higher brightness of the mesh element defines a bigger probability of such an element belonging to the inclusion. It can be noted that the result with ridge or LASSO regularization roughly defines the position and shape of the inclusion.

However, the result for ridge regularization is reasonably good and represents the object’s center. The better result gives a combination of both methods. We observe visible image quality enhancement. In this result, a constriction of the inclusion area towards the center of the inclusion can also be noted.

**Figure 8:**
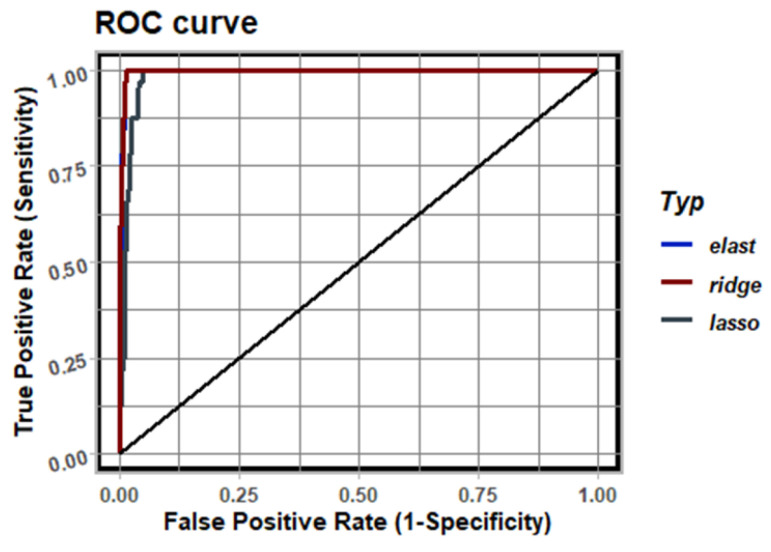
ROC analysis for results shown in Figure 7.

Figure 8 shows the ROC curve for the results in Figure 7 for the given regularization methods. It can be seen that the lines for elastic net and ridge regularization overlap relatively well, where the ROC curve for LASSO differs from the other two. This result shows that the best fit is obtained by ridge regularization, and it is the main component of the discretization classifier that defines its diagnostic ability.

Table 2 shows the values of the basic coefficients, Cohen’s ratio, and McNemar’s test for LR and every regularization method. The κ ratio shows that the highest credibility of performed classifications is obtained by the LR method with Elastic Net regularization. On the other hand, the smallest $\chi^{2}$ inconsistency between the snapshot and discretization is also performed using this method.

### 4.2. Results for Linear and Quadratic Discriminant Methods

**Figure 9:**
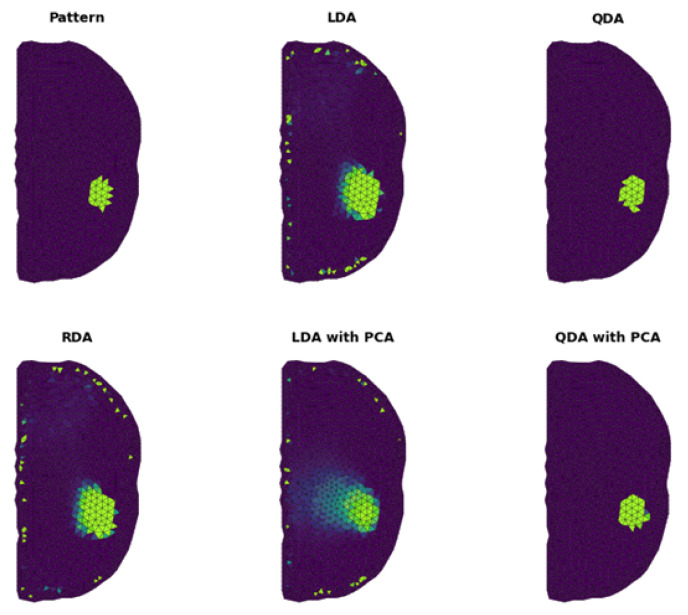
The top left panel shows the shape of the inclusion. The other panels show the results obtained by the discriminant method with/without dimensionality reduction.

Figure 9 shows the results obtained for the discriminant methods without and with applied dimensionality reduction. The top left panel in Figure 9 represents the inclusion pattern. The next panels show the results for LDA, QDA and RDA, respectively. It can be noted that the predictions of those classifiers indicate the position of the inclusion very well. Additionally, the QDA method almost perfectly illustrates the shape of the predicted object.

**Figure 10:**
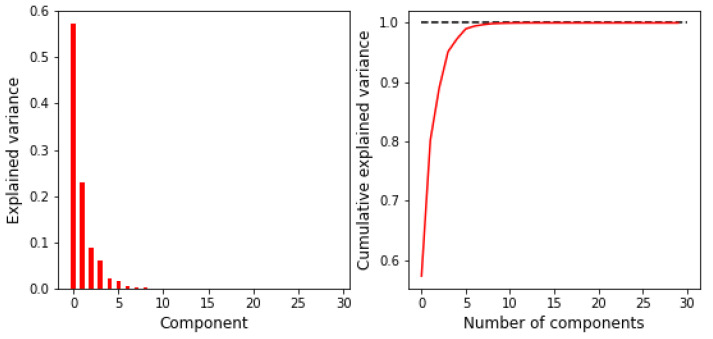
Principal component analysis explained variance plots. The (**left**) panel shows the percentage of explained variance by particular components. The (**right**) panel presents the cumulative variance explained by the first *k* components.

The presence of noise can also be noted, with a randomly higher probability for single elements near the mesh boundary in the result for the LDA and RDA methods. The last two panels in Figure 9 represent the LDA and QDA methods with applied principal components analysis (PCA). In the result, we observe the improvement of the discretization quality for LDA, where the QDA application of the PCA does not significantly affect the final probability distribution. Comparing the results in Figure 9, one can notice that the QDA with PCA is the best match of the prediction with an assumed inclusion pattern qualitatively. A closer look at PCA, presented in Figure 10, reveals that the first eight principal components can fully explain the variance.

**Figure 11:**
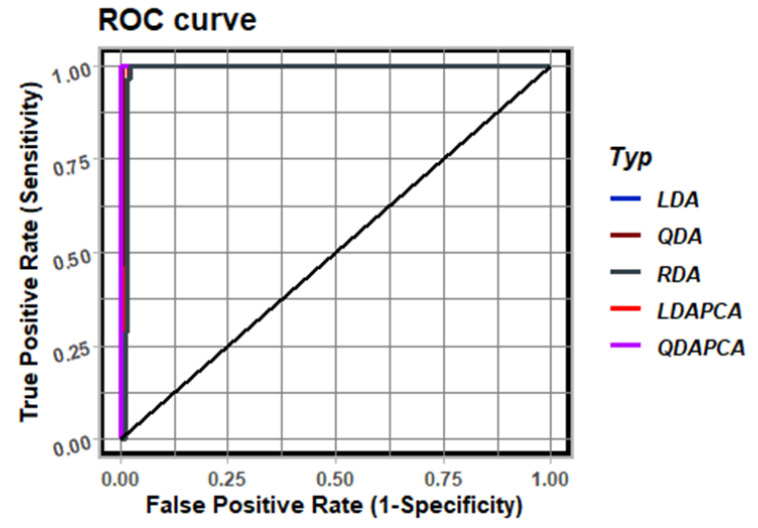
ROC analysis for results shown in Figure 9.

In Figure 11, we show the ROC curve for the results presented in Figure 7, including the dimensionality reduction method. Note that all of the used approaches result in the high-quality discretization of FEM components. This graph shows that the RDA has the lowest diagnostic ability compared with the other methods.

In Table 3, we present the fit measures for every discriminant method. The κ value indicates that QDA obtains the best result with the applied PCA. However, the lowest inconsistency $\chi^{2}$ is provided by QDA without the application of PCA. This inconsistency is visible in Figure 9, where the modest difference between the result of QDA and QDA with applied PCA can be noted, resulting in extra mesh elements (classified as the object) at the bottom of the predicted inclusion.

### 4.3. Results for Classification and Regression Trees

**Figure 12:**
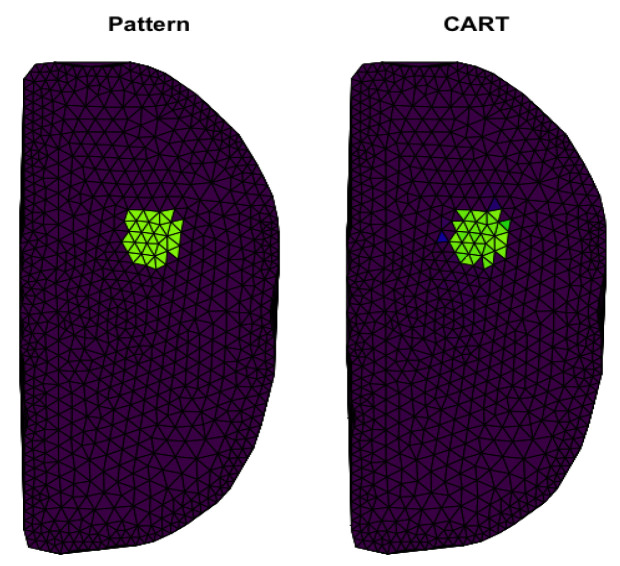
The first panel presents the inclusion shape. The second panel shows reconstruction obtained by CART.

In Figure 12, we present the use of regression trees in our classification problem. This result clearly shows the high alignment between reconstruction and inclusion pattern; we can observe almost a one-to-one mapping. In this example, we can notice particular elements incorrectly classified as pattern elements. However, comparing this graph with the results obtained by previous methods, it can be seen that the decision trees algorithm shows the best reconstruction quality. Since CART is robust to collinearity, none of the previously mentioned methods of regularization or dimensionality reduction are needed.

**Figure 13:**
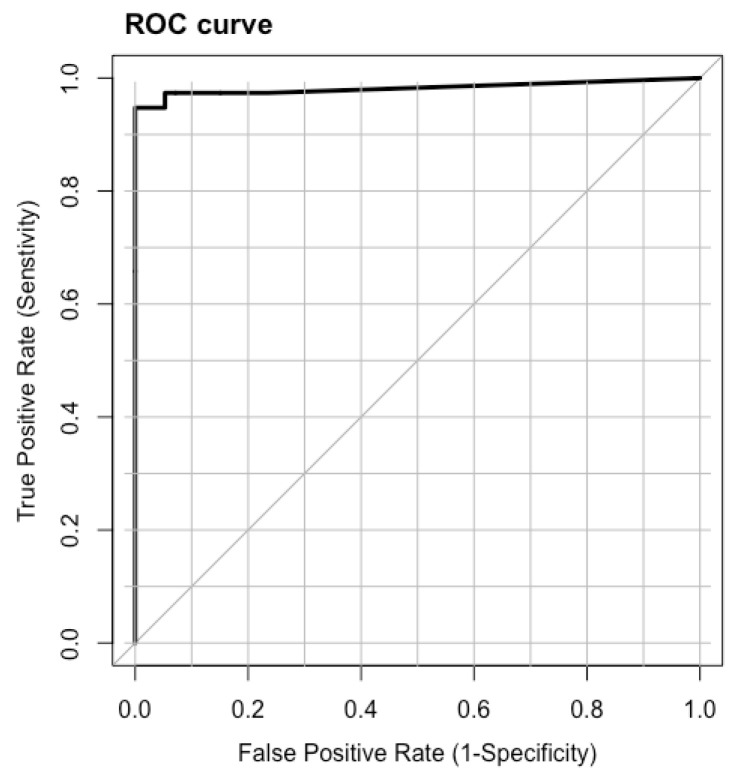
ROC analysis for results shown in Figure 12.

In detail, Figure 13 shows the ROC curve for the results presented in Figure 12. This result indicates high-quality discretization of FEM components.

In Table 4, we present the values of basic coefficients for the decision trees method. The value of the κ ratio confirms the highest accuracy of the reconstruction, and the small inconsistency defined by $\chi^{2}$ is also achieved.

### 4.4. Reconstruction Performance

In order to assess the reconstruction performance, the following measures were used:

(35) $$MSE=\frac{1}{nm}\sum_{i=0}^{m-1}\sum_{j=0}^{n-1}{∥I\left(i,j\right)-K\left(i,j\right)∥}^{2},$$

(36) $$MAE=\frac{1}{nm}\sum_{i=0}^{m-1}\sum_{j=0}^{n-1}∥I\left(i,j\right)-K\left(i,j\right)∥,$$

(37) $$PSNR=10\;\cdot \;\log_{10}\frac{{\left(\max x_{i}\right)}^{2}}{MSE},$$

(38) $$SSIM=\frac{\left(2\mu_{I}\mu_{K}+c_{1}\right)\left(2\sigma_{IK}+c_{2}\right)}{\left(\mu_{1}^{2}+\mu_{2}^{2}+c_{1}\right)\left(\sigma_{1}^{2}+\sigma_{2}^{2}+c_{2}\right)},$$

where $\mu_{I},\mu_{K}$ are means of *I*, *K*; $\sigma_{1}^{2},\sigma_{2}^{2}$ are variances of *I*, *K*; $\sigma_{IK}$ is the covariance matrix of *I*, *K*; $c_{1}$, $c_{2}$ are some constants; and *I* i *K* are the images of N×N. Assuming that I(i,j) and K(i,j) are values of (i,j) pixel in the original and reconstructed image *I*, *K*, respectively. These are well-known metrics used in assessing reconstruction quality [28,29,30].

In Table 5, we present the values of measures for the introduced algorithms and compare them with the values for selected literature models. Reconstructions obtained using different machine learning techniques produce different results in the context of the adopted measures of reconstruction quality. The best reconstructions were obtained using decision trees (all measures have the best results). QDA and QDAPCA methods give slightly worse results in the context of all analyzed measures. The worst results were obtained using ridge regression and LASSO regularization models.

Comparing the results with those obtained in other works [31,32,33,34,35,36,37,38] in the field of EIT reconstruction using machine learning methods, it can be concluded that at least some of the models presented in this work (especially the CART model) dominate the published achievements in terms of the obtained measures of reconstruction quality.

The best of our models (CART) outperforms most models in the literature in terms of the PSNR measure, except for TN-Net, which has a slightly higher value. In contrast, none of the published models tops our models in terms of the SSIM measure. The less frequently used MSE measure, on the other hand, shows that some models such as DNN, CNN and TN-Net are better than most of our models except for the CART model, which also dominates in terms of this measure.

### 4.5. Application of the Algorithms to a Real Case

**Figure 14:**
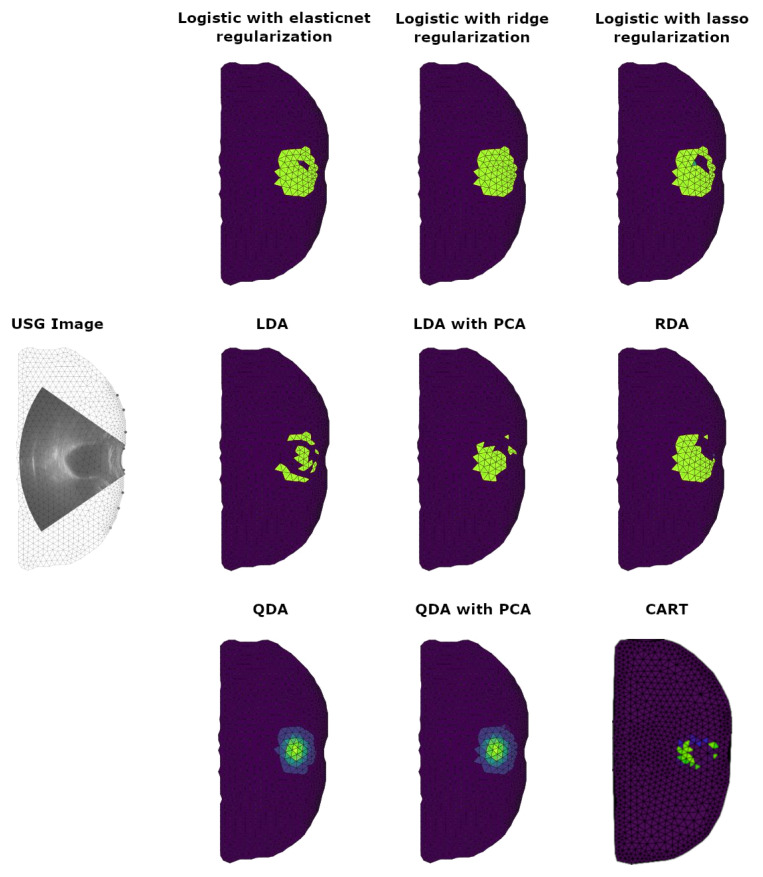
Comparison of results obtained for real measurement data using introduced discretization methods. As a reference, we show the USG image for the bladder superimposed on the finite element mesh with the proportions maintained.

The comparison of the results obtained using the developed discretization methods with the actual measurement data is shown in Figure 14. The first row contains results for logistic regression with regularization methods, the second row contains LDA, LDA with PCA and RDA, and the last row contains data for QDA, QDA with PCA and CART. These results show that a finite element mesh represents the probability distribution of finding a bladder in the abdominal cavity.

In the results, it can be seen that some methods do not give sufficient results due to the discontinuity of the reconstructions and the irregularity of their shape. For example, the logistic regression method gives a reconstruction with an oval shape of the studied object, while elastic net and LASSO regularization result in a hollow area inside the bladder. Moreover, it can be noted that the object reconstructed by logistic regression is too close to the abdominal wall, which is biologically impossible due to the presence of abdominal tissue.

The second row of the results presented in Figure 14 shows that the LDA method does not give reliable reconstructions regardless of regularisation. We observe that the reconstruction does not create a uniform structure and is divided into many fragments. It discredits the algorithm as a reliable source of information. Similar behavior is observed with RDA.

The last line in Figure 14 contains the results for QDA, QDA with PCA and CART. The sensitivity of QDA methods allows to sufficiently capture the shape of the bladder. Due to its real shape, it can be expected that the probability distribution should be larger in the center of the object and smaller outside. This result is clearly visible in the reconstruction plots. Contrary to the good quality of reconstructions for QDA methods, the results obtained with the CART method are far from acceptable. We observe that the reconstruction obtained with CART yields several separate regions. It is quite surprising because the CART algorithm has a very high efficiency of reconstruction obtained from simulated measurements (see Table 4).

## 5. Discussion

The main objective of our study is to find the best classifier for discrete elements of the introduced FEM and real measurements. In this work, logistic regression, linear discriminant analysis, quadratic discriminant analysis and decision trees were used to reconstruct the inclusion image using the signal obtained from the simulation. In the next step, those models were used to reconstruct the image of the bladder using a real data frame. Since some of the methods are sensitive to redundancy, various types of regularization and dimensionality reductions were applied. To give an answer to the question of which method gives the best reconstruction, we show the results obtained for random simulated inclusion patterns and collected measurement data. In order not to rely only on visual data, many of the fit measures were estimated: accuracy, sensitivity, specificity, positive predictive value, negative predictive value, detection rate, and AUC. Furthermore, tests of the reliability of the reconstruction (Cohen’s kappa) and the discrepancy between the model and the reconstruction (McNemar’s test) were performed.

Comparing such characteristics as accuracy (the part of the field of view that the model correctly recognized as inclusion) and specificity (the part of the field of view belonging to the background), we see that their values are quite accurate for all of the studied methods. However, the visual analysis of the reconstructions indicates that reconstruction quality drastically differs between the methods. The differences above stand out when comparing positive predicted value, negative predicted value, precision, and F1 measures between models. The results for LR and LDA do not provide sufficient good reconstructions. On the other hand, QDA with dimensionality reduction and decision trees present a very good fit in this context.

To select the best qualitative results, we have to rely on more representative coefficients, i.e., Cohen’s coefficient and McNemar’s test. Comparing the results for κ and $\chi^{2}$, one can notice that the best results provide the QDA method and decision trees. Comparing models with regularization and dimensionality reduction with models without redundancy correction clearly shows that the former has a better fit. To determine the final reliability of the models, we compare their predictions gained from the real measurements. We observe a significant advantage in the quality of reconstructions obtained by the QDA method over decision trees.

## 6. Conclusions

The presented monitoring system is designed for automatic and unsupervised bladder tracking using EIT. Besides tracking, this solution allows for bladder imaging using the inverse problem solution. In this work, we focused on adapting statistical methods for the problem of bladder discretization together with FEM representing the abdomen cross section at the bladder level. We introduced several methods, such as logistic regression (LR), linear and quadratic discriminant methods (LDA, QDA) and decision trees. For LR, LDA and QDA, we implemented several regularization methods, e.g., ridge, LASSO and PCA.

The underlying reason for our research was the implementation of the algorithm for tracking the bladder, disregarding the problem of the impedance distribution in the interior of the studied object. The reliable algorithms for high prediction accuracy provide the ability to track the bladder, as well as the ability to monitor its filling. In addition, those algorithms could work in hybrid mode together with deterministic methods for the inverse problem to obtain the high-resolution impedance distribution in the studied case. In our work, we placed the measuring electrodes on only one side of the patient’s abdomen. This approach opens up new possibilities for creating friendlier and easier-to-use monitoring devices, which are more comfortable for the patient to wear.

In conclusion, we have shown that logistic regression is not sufficient for our task, despite the use of the regularization method. The results obtained by LR are strongly distorted. The reconstruction roughly determines the center of the object but completely loses information about the shape of the inclusion. The better performance was the discrimination result obtained by the LDA method, where the position and shape of the prediction are fairly well-defined. However, the LDA method does not achieve enough high accuracy required for bladder tracking. In our study, the finest and most trustworthy results are given by QDA, characterized by a high Cohen ratio and minor inconsistency $\chi^{2}$ defined by McNemar’s test.

Thus, the presented study results contain significant information that may accelerate the development of bladder tracking methods in medical tomography. In addition, the research contributes to improving the accuracy of tomographic imaging. The presented algorithm can be used as a hybrid method for predicting an object’s electrical properties.

## Figures and Tables

**Table 1:** Confusion matrix.

	Positive	Negative
Positive Prediction	*TP*	*FP*
Negative Prediction	*FN*	*TN*

**Table 2:** Table of fit measures obtained for the reconstruction shown in Figure 7.

	Elastic Net	Ridge	LASSO
Accuracy	0.900	0.889	0.857
Sensitivity	1.000	1.000	1.000
Specificity	0.898	0.887	0.854
Pos Pred Value	0.170	0.157	0.126
Neg Pred Value	1.000	1.000	1.000
Precision	0.170	0.157	0.126
F1	0.291	0.271	0.224
Prevalence	0.021	0.021	0.021
Detection Rate	0.021	0.021	0.021
Detection Prevalence	0.121	0.131	0.163
Balanced Accuracy	0.949	0.944	0.927
AUC	0.996	0.997	0.985
κ	0.265	0.244	0.194
$\chi^{2}$	154.006	170.006	220.005

**Table 3:** Table of fit measures obtained for the reconstruction shown in Figure 9.

	LDA	QDA	RDA	LDAPCA	QDAPCA
Accuracy	0.943	0.995	0.947	0.947	0.995
Sensitivity	1.000	0.929	1.000	1.000	1.000
Specificity	0.942	0.996	0.946	0.946	0.995
Pos Pred Value	0.241	0.813	0.252	0.252	0.778
Neg Pred Value	1.000	0.999	1.000	1.000	1.000
Precision	0.241	0.812	0.252	0.252	0.778
F1	0.389	0.867	0.403	0.403	0.875
Prevalence	0.018	0.018	0.018	0.018	0.018
Detection Rate	0.018	0.017	0.018	0.018	0.018
Detection Prevalence	0.075	0.021	0.071	0.071	0.023
Balanced Accuracy	0.971	0.962	0.973	0.973	0.997
AUC	0.986	0.998	0.985	0.988	1.000
κ	0.371	0.864	0.385	0.385	0.872
$\chi^{2}$	86.011	1.125	81.012	81.012	6.125

**Table 4:** Table of coefficients obtained for the reconstruction shown in Figure 12.

	CART
Accuracy	0.999
Sensitivity	0.947
Specificity	1.000
Pos Pred Value	1.000
Neg Pred Value	0.999
Precision	1.000
F1	0.973
Prevalence	0.023
Detection Rate	0.022
Detection Prevalence	0.022
Balanced Accuracy	0.973
AUC	0.982
κ	0.972
$\chi^{2}$	2

**Table 5:** Table of measures that determine the quality of the reconstructions.

Model	MAE	MSE	SSIM	PSNR
Elastic	0.11	0.05	1.00	12.53
Ridge	0.15	0.05	1.00	10.89
LASSO	0.14	0.10	1.00	9.99
LDA	0.06	0.05	1.00	13.22
QDA	0.01	0.01	1.00	22.93
RDA	0.06	0.05	1.00	13.40
LDA PCA	0.07	0.03	1.00	14.84
QDA PCA	0.01	0.01	1.00	23.00
CART	0.00	0.00	1.00	28.92
DNN [31]	-	0.0083	-	-
CNN [32]	-	0.0140	0.9011	18.5387
MMV-Net [33]	-	0.049	0.9354	23.7423
Kernel method [34]	-	-	0.7822	-
En-MSFCF-Net [35]	-	-	0.9862	-
RCRC [36]	-	33.177	0.68	-
TN-Net [37]	-	0.0058	0.9657	30.709
VDD-Net [38]	-	0.941	-	-

## Data Availability

Not applicable.
